# Comparison of two surgical approaches for acute type A aortic dissection: hybrid debranching versus total arch replacement

**DOI:** 10.1186/s13019-022-01920-9

**Published:** 2022-06-23

**Authors:** Feng Huang, Xiaofeng Li, Zili Zhang, Chunping Li, Fei Ren

**Affiliations:** 1 Department of Cardiovascular Surgery, Fujian Provincial Hospital, Fuzhou, 350001 China; 2 Shengli Clinical Medical College of Fujian Medical University, Fuzhou, 350001 China; 3 Department of the Third Internal Medicine, Anqiu Municipal Hospital, Weifang, 262100 China; 4 Department of Cardiovascular Surgery, Fujian Provincial Geriatric Hospital, Fuzhou, 350000, China

**Keywords:** Acute type A aortic dissection, Hybrid debranching, Total arch replacement, Clinical outcomes

## Abstract

**Background:**

The goal of this study was to determine the clinical outcomes of total arch replacement with frozen elephant trunk surgery and hybrid debranching surgery for acute type A aortic dissection patients.

**Methods:**

From January 2017 to December 2019, the clinical data of acute type A aortic dissection patients were retrospectively collected and analyzed. There were 142 patients underwent total arch replacement with frozen elephant trunk surgery and 35 patients underwent hybrid debranching surgery.

**Result:**

The age, the body mass index and the renal insufficiency of patients in the hybrid group were higher than those in the total arch replacement (TAR) group (all *P* < 0.01). The operation time, the cardiopulmonary bypass time and the aortic occlusion time of patients in the TAR group were significantly longer than those in the hybrid group (all *P* < 0.01). Patients in the debranching group had shorter ventilator-assisted breathing time, shorter postoperative hospital stay time and shorter intensive care unit (ICU) stay time. The incidence of pulmonary infection and transient neurological dysfunction were lower, and the transfusions of red blood cells and plasma during the perioperative period were smaller. The survival rates at 2 years were 91.9% and 85.9% in the TAR and hybrid groups, respectively.

**Conclusion:**

Hybrid debranching operation is a safe and effective method for acute type A aortic dissection. Compared with TAR surgery, hybrid debranching surgery has the characteristics of less trauma, rapid recovery and lower incidence of complication.

## Introduction

Aortic dissection is a cardiovascular disease with a high mortality rate, with an hourly mortality rate of 1 to 2% for untreated patients [1]. Even after emergent surgical repair, the International Registry of Acute Aortic Dissection cites nearly 20% surgical mortality and more than 30% in-hospital mortality for Type A Aortic Dissection [2]. In particular, for acute type A aortic dissection, the breach is located in the ascending aorta and involves the aortic arch or its distal end. The lesion involves a wide range and may involve important blood vessels, such as the celiac trunk, superior and inferior mesenteric arteries, left and right renal arteries, and left and right iliac arteries. Therefore, emergency surgical treatment should be performed immediately after diagnosis [3].

Total arch replacement combined with stented frozen elephant trunk implantation has significantly improved the therapeutic effect of aortic arch lesions. With the extensive application and improvement of stented frozen elephant trunk implantation, the mortality rate of patients has decreased year by year, but it still has several disadvantages, including severe surgical trauma and deep hypothermic circulatory arrest [4]. With the maturity of vascular stent technology, hybrid surgery came into being, which has the characteristics of less trauma and rapid recovery [5]. Hybrid debranching surgery and total arch replacement (TAR) surgery are the possible choices for acute type A aortic dissection, but the results may be different due to different surgical methods. Here, the goal of this article is to compare the clinical data of Hybrid debranching surgery and TAR surgery, to provide a reference for patients' choice of surgical methods.

## Patients and methods

### Patients

We retrospectively analyzed patients who underwent TAR surgery or Hybrid debranching surgery for acute type A aortic dissection at our hospital from January 2017 to December 2019. We excluded patients with chronic aortic dissection, pregnant, aortic dissection caused by trauma, Marfan syndrome and secondary surgery. Therefore, a total of 177 patients were included in the study. 142 of these patients underwent TAR surgery, and 35 underwent hybrid surgery. In this study, whole aortic computed tomography angiography (CTA) was used as the diagnostic gold standard, and all patients received a total aortic CTA. In this study, TAR is the preferred treatment option for acute type A aortic dissection. We also selected hybrid surgery for high-risk patients based on their age, preoperative status and high-risk factors.

### Surgical procedure

#### Hybrid debranching procedure

After a median sternotomy, the right axillary artery, femoral artery and right atrial were cannulated to establish cardiopulmonary bypass. An occluding clamp was applied to block the ascending aorta, and the nasopharyngeal temperature was gradually cooling. The surgical methods for aortic root were determined according to the type of lesion at the proximal end of the aorta, including transposition of ascending main artery, Bentall, Wheat, David, etc. The proximal end of the trimmed four-branch artificial blood vessel was anastomosed with the ascending aorta by continuous suture, and the distal end of the artificial blood vessel was anastomosed with the ascending aorta at the proximal end of the innominate artery. The ascending aortic clamp was opened, and the circulation was restored. The branches of the artificial blood vessel were anastomosed with the left subclavian artery, the left common carotid artery and the innominate artery, respectively. A silver clip was marked at the distal anastomosis of the artificial blood vessel. The superhard guidewire was placed through the femoral artery with the pigtail catheter, and the aortic stent was put through the superhard guidewire. The proximal end of the stent was 2 cm beyond the distal anastomosis of the artificial vessel, then the stent (Microport, Shanghai, China)was released after blood pressure was lowered.

#### Total arch replacement procedure

**Fig. 1:**
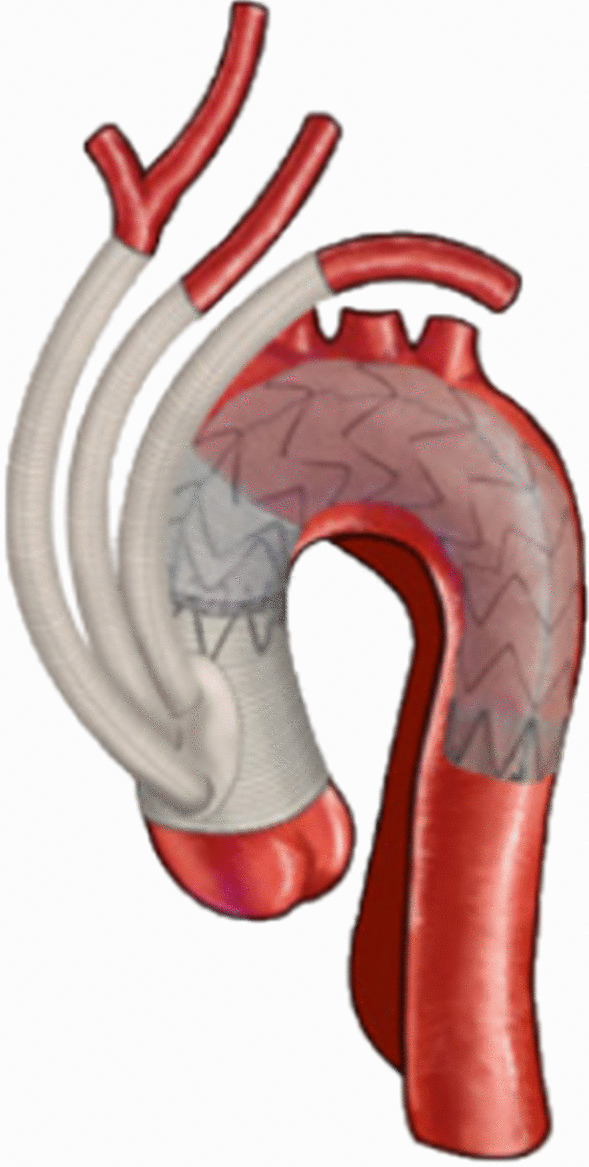
Hybrid debranching procedure

**Fig. 2:**
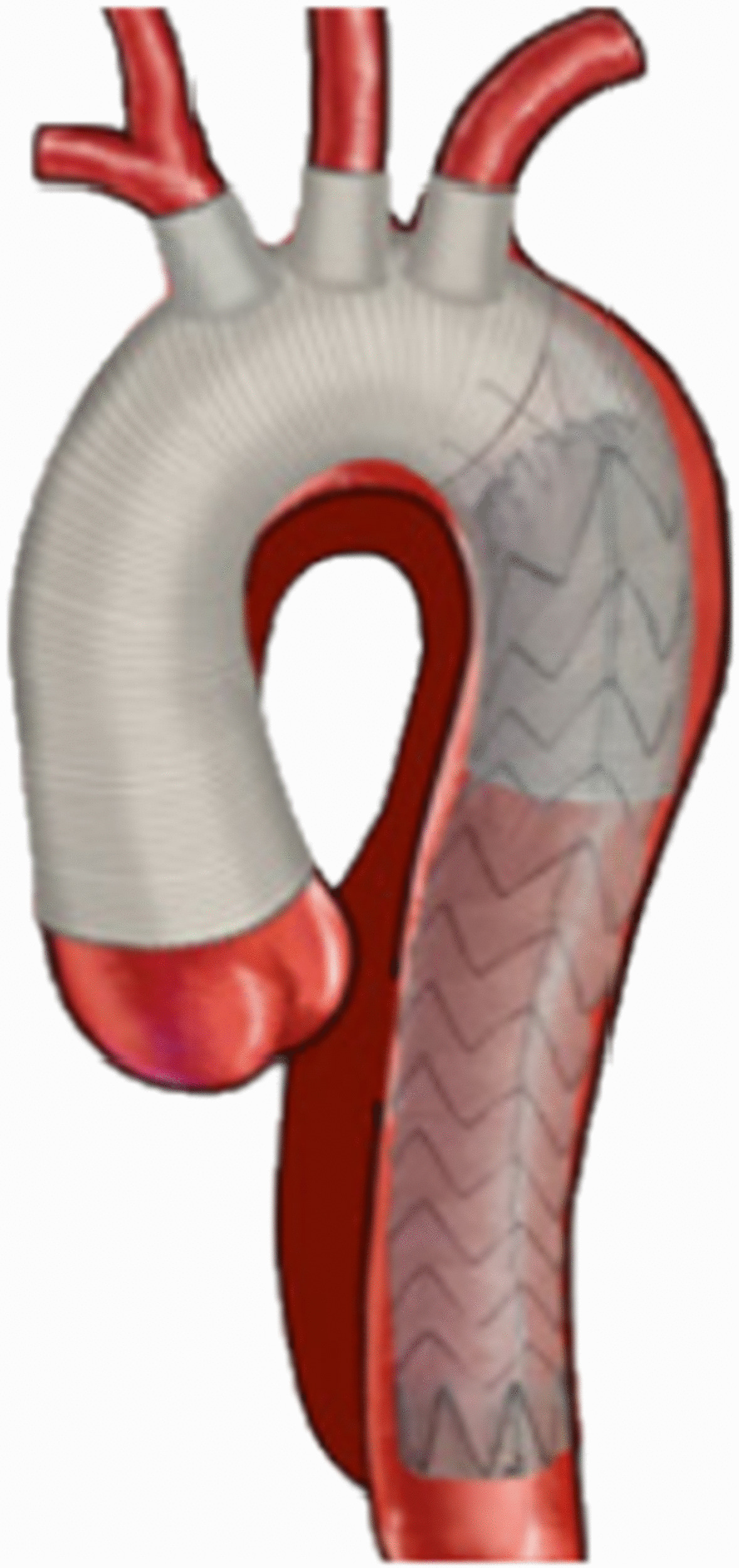
Total arch replacement procedure

After a median sternotomy, the right axillary artery, femoral artery and right atrial were cannulated to establish cardiopulmonary bypass. An occluding clamp was applied to block the ascending aorta, and the nasopharyngeal temperature was gradually cooling. The surgical methods for aortic root were determined according to the type of lesion at the proximal end of the aorta, including transposition of ascending main artery, Bentall, Wheat, David, etc. When the nasopharyngeal temperature dropped to 20–24 °C, circulation was stopped, and anterograde cerebral perfusion from the right axillary artery was performed. The aorta was opened longitudinally in the aortic arch to clear the thrombus. The stent elephant trunk was implanted in the descending aorta. The proximal end of the stent elephant trunk and the descending aortic wall were anastomosed with the distal end of four branches artificial blood vessel. The blood is infused into the lower body through four branches artificial blood vessel, and the rewarming is followed. During rewarming, the brachiocephalic trunk artery and the left common carotid artery are anastomosed with the branches of artificial blood vessel, respectively. After the aortic arch was reconstructed, the ascending aorta was anastomosed with the four-branch artificial blood vessel. Finally, the left subclavian artery is anastomosed with another branch of the artificial blood vessel (Figs. 1, 2).

### Postoperative treatment

When the patients were transferred to the ICU after surgery, ventilators were applied to assist breathing, and the vital signs were closely monitored. After being disconnected from the ventilator, patients were transferred to the general ward if they had stable hemodynamic parameters and no severe complications (pulmonary infection, impaired consciousness, limb mobility, cardiac failure, etc.). Patients with mechanical valve replacement require warfarin for life time, and regular testing of prothrombin time (PT) and international standardized ratio (INR). Aortic CTA was reexamined in 3 months and 12 months after operation, and then once a year. All the patients were followed up by outpatient or telephone, to assess survival and reintervention.

### Data collection and follow-up

The preoperative data included age, sex, body mass index, coronary heart disease, hypertension, diabetes, smoking history, liver function, renal function, and cardiac colour doppler index (left ventricular ejection fraction, Ascending Aortic diameter, Aortic sinus diameter). The intraoperative data included aortic cross-clamp time, cardiopulmonary bypass (CBP) time, circulatory arrest time and the operation time. Postoperative data included hospital stay, ICU-stay, mechanical ventilation time, blood transfusions, renal failure, neurological dysfunction, pulmonary infection, and perioperative mortality. All the patients were followed up every 6 months. The follow-up data included survival status and reviews of CTA.

### Statistical analysis

All the data were statistically analyzed using SPSS 22.0 software. We applied propensity score match with a 1:1 matching algorithm, using ± 0.15 caliper that did not require replacement to adjust for significantly different patient characteristics. This analysis resulted in 35 observations of propensity score matching. We compared the standardized mean differences of all covariates after matching. Measurement data were expressed as mean ± standard deviation, and inter-group comparison was performed using independent sample t test. Enumeration data were expressed as case numbers and percentages, and inter-group comparisons were performed using the χ2 test or Fisher exact probability method. Kaplan–Meier- method was used for survival analysis. The difference was statistically significant (*P* < 0.05).

## Result

### Comparison of preoperative data

Preoperative patient characteristics are summarized in Table 1. The age(53.9 ± 6.8 vs 62.1 ± 6.17 years), the body mass index (24.8 ± 1.5 vs 25.8 ± 1.6 kg/m^2^) and the renal insufficiency(0%vs14.3%) of patients in the hybrid group were higher than those in the TAR group (all *P* < 0.01).

**Table 1:** Preoperative data

Variables	Total cohort (n = 177)			Propensity-matched cohort (n = 70)		
	TAR	Hybrid	*P* value	TAR	Hybrid	*P* value
	(n = 142)	(n = 35)		(n = 35)	(n = 35)	
Male, n (%)	116 (81.7)	23 (65.7)	0.068	23 (65.7)	23 (65.7)	1
Age (y)	53.9 ± 6.8	62.1 ± 6.2	< 0.001	62.5 ± 6.8	62.1 ± 6.2	0.746
Hypertension, n (%)	129 (90.8)	32 (91.4)	0.983	31 (88.6)	32 (91.4)	0.983
Diabetes, n (%)	54 (38.0)	12 (34.3)	0.93	12 (34.3)	12 (34.3)	1
CHD, n (%)	24 (16.9)	8 (22.9)	0.461	7 (20.0)	8 (22.9)	1
Smoking, n (%)	40 (28.2)	12 (34.3)	0.519	10 (28.6)	12 (34.3)	0.819
BMI, (kg/m^2^)	24.8 ± 1.5	25.8 ± 1.6	0.002	25.1 ± 1.5	25.8 ± 1.6	0.322
Liver dysfunction, n (%)	3 (2.11)	3 (8.6)	0.069	3 (8.6)	3 (8.6)	1
Renal dysfunction, n (%)	2 (2.1)	5 (14.3)	0.006	4 (11.4)	5 (14.3)	1
Ejection fraction (%)	57.5 ± 2.9	57.8 ± 2.6	0.635	57.5 ± 2.9	57.8 ± 2.6	0.635
AAD (mm)	46.4 ± 7.1	47.5 ± 4.8	0.329	47.4 ± 5.1	47.5 ± 4.8	0.729
ASD (mm)	38.5 ± 6.0	37.4 ± 4.6	0.344	38.5 ± 6.0	37.4 ± 4.6	0.344
Hydropericardium, n (%)	7 (4.9)	4 (11.4)	0.288	3 (8.6)	4 (11.4)	1
AVI, n (%)	49 (34.5)	6 (17.1)	0.073	8 (22.9)	6 (17.1)	0.873

Y, years; mm, millimeter; TAR, total arch replacement; CHD, coronary artery heart disease; BMI, body mass index; AAD,aorta arch diam; ASD, aorta ascendens diam; AVI, aortic valve insufficiency

There were no significant differences in sex, hypertension, diabetes mellitus, coronary heart and liver dysfunction, smoking cigarettes, and indexes of cardiac color ultrasound between the two groups. After propensity score match, there was no significant difference in basic conditions and clinical risk factors between the two groups.

### Comparison of intraoperative data

The intraoperative data of the patients are shown in Table 2.

**Table 2:** Intraoperative data

Variables	Total cohort (n = 147)			Propensity-matched cohort (n = 70)		
	TAR	Hybrid	*P* value	TAR	Hybrid	*P* value
	(n = 142)	(n = 35)		(n = 142)	(n = 35)	
Operation time (h)	8.4 ± 1.3	6.5 + 0.8	< 0.001	8.2 ± 1.45	6.5 + 0.8	< 0.001
CBP time (min)	226 ± 27.7	142.1 ± 20.6	< 0.001	232.3 ± 20.2	142.1 ± 20.6	< 0.001
ACC time (min)	127.6 ± 15.2	95.5 ± 14.6	< 0.001	131.3 ± 18.7	95.5 ± 14.6	< 0.001
AC time (min)	39.0 ± 5.1	0	< 0.001	37.8 ± 9.3	0	< 0.001

h, hour; min, minute; CBP, cardiopulmonary bypass; ACC, aortic cross-clamp; CA, circulatory arrest; TAR, total arch replacement

Both before and after matching, the operation time, the cardiopulmonary bypass time and the aortic occlusion time of patients in the TAR group were significantly longer than those in the hybrid group (all *P* < 0.001). In the unmatched cohort, the time of deep hypothermic circulatory arrest in TAR operation was 39.0 ± 5.1 min, but the time of deep hypothermic circulatory arrest in hybrid debranching operation was 0 min. In the propensity-matched cohort, the time of deep hypothermic circulatory arrest in TAR operation was 37.8 ± 9.3 min, but the time of deep hypothermic circulatory arrest in hybrid debranching operation was 0 min.

### Comparison of postoperative data

The postoperative data of the patients are shown in Table 3.

**Table 3:** Postoperative data

Variables	Total cohort (n = 147)			Propensity-matched cohort (n = 70)		
	TAR	Hybrid	*P* value	TAR	Hybrid	*P* value
	(n = 142)	(n = 35)		(n = 35)	(n = 35)	
ICU time (d)	7.4 ± 2.0	2.8 ± 1.0	< 0.001	6.8 ± 2.1	2.8 ± 1.4	< 0.001
Ventilation time(h)	63.5 ± 19.7	18.5 ± 9.2	< 0.001	59.7 ± 21.2	18.5 ± 9.2	< 0.001
Hospital stay time(d)	17.9 ± 4.7	12.3 ± 4.3	< 0.001	19.5 ± 6.4	12.3 ± 4.3	< 0.001
Erythrocyte volume(u)	6.6 ± 2.4	4.2 ± 0.7	< 0.001	4.5 ± 3.1	4.2 ± 0.7	0.103
Plasma volume(ml)	511.3 ± 190.1	420.0 ± 211.2	0.027	421 ± 152.1	420.0 ± 211.2	0.512
Pulmonary infection, n (%)	21 (29.6)	4 (11.4)	0.038	7 (20.0)	4 (11.4)	0.328
Renal dysfunction, n (%)	10 (14.1)	3 (8.57)	0.416	6 (17.1)	3 (8.57)	0.912
TND, n (%)	19 (26.8)	3 (8.6)	0.03	3 (8.6)	3 (8.6)	1
PND, n (%)	4 (5.6)	0	0.152	3 (8.6)	0	0.752
30-Day mortality, n (%)	6 (8.5)	1 (2.9)	0.268	4 (11.4)	1 (2.9)	0.738

h, hour; d, day; u, unit; ml, milliliter; TND, Transient neurological dysfunction; PND, permanent neurological dysfunction; TAR, total arch replacement; ICU, intensive care unit

During hospitalization, six patients (8.5%) died in the TAR group, including 3 patients due to multiple organ failure, one patient due to severe infection, one patient due to cerebral infarction, and one patient due to paraplegia. There was one patient (2.9%) in the debranching group died as a result of multiple organ failure.

In the unmatched cohort, patients in the debranching group had shorter ventilator-assisted breathing time (63.5 ± 19.7 vs 18.5 ± 9.2 h, *P* < 0.001), shorter postoperative hospital stay time (17.9 ± 4.7 vs 12.3 ± 4.3 days, *P* < 0.001) and shorter ICU stay time (7.4 ± 2.0 vs 2.8 ± 1.0 days < 0.001). The incidence of pulmonary infection (29.6% vs 11.4%, *P* = 0.038) and transient neurological dysfunction (26.8% vs 8.6%, *P* = 0.030) were lower, and the transfusions of erythrocyte (6.6 ± 2.4 vs 4.2 ± 0.7 units, *P* < 0.001) and plasma (511.3 ± 190.1 vs 420.0 ± 211.2 ml, *P* = 0.027)during the perioperative period were smaller. No statistically significant differences were observed in Perioperative mortality (*P* = 0.268), Renal dysfunction (*P* = 0.416), Permanent neurological dysfunction (*P* = 0.152).

In the propensity-matched cohort, the ventilator time (59.7 ± 21.2 vs 18.5 ± 9.2 h, *P* < 0.001), the hospital time (19.5 ± 6.4 vs 12.3 ± 4.3 days, *P* < 0.001) and the ICU time (6.8 ± 2.1 vs 2.8 ± 1.0 days < 0.001) in the Hybrid group were shorter than that in the TAR group. There were no significant differences between the 2 groups in terms of Plasma volume, erythrocyte volume, renal dysfunction, TND, PND and 30-Day mortality.

### Comparison of follow-up data

**Fig. 3:**
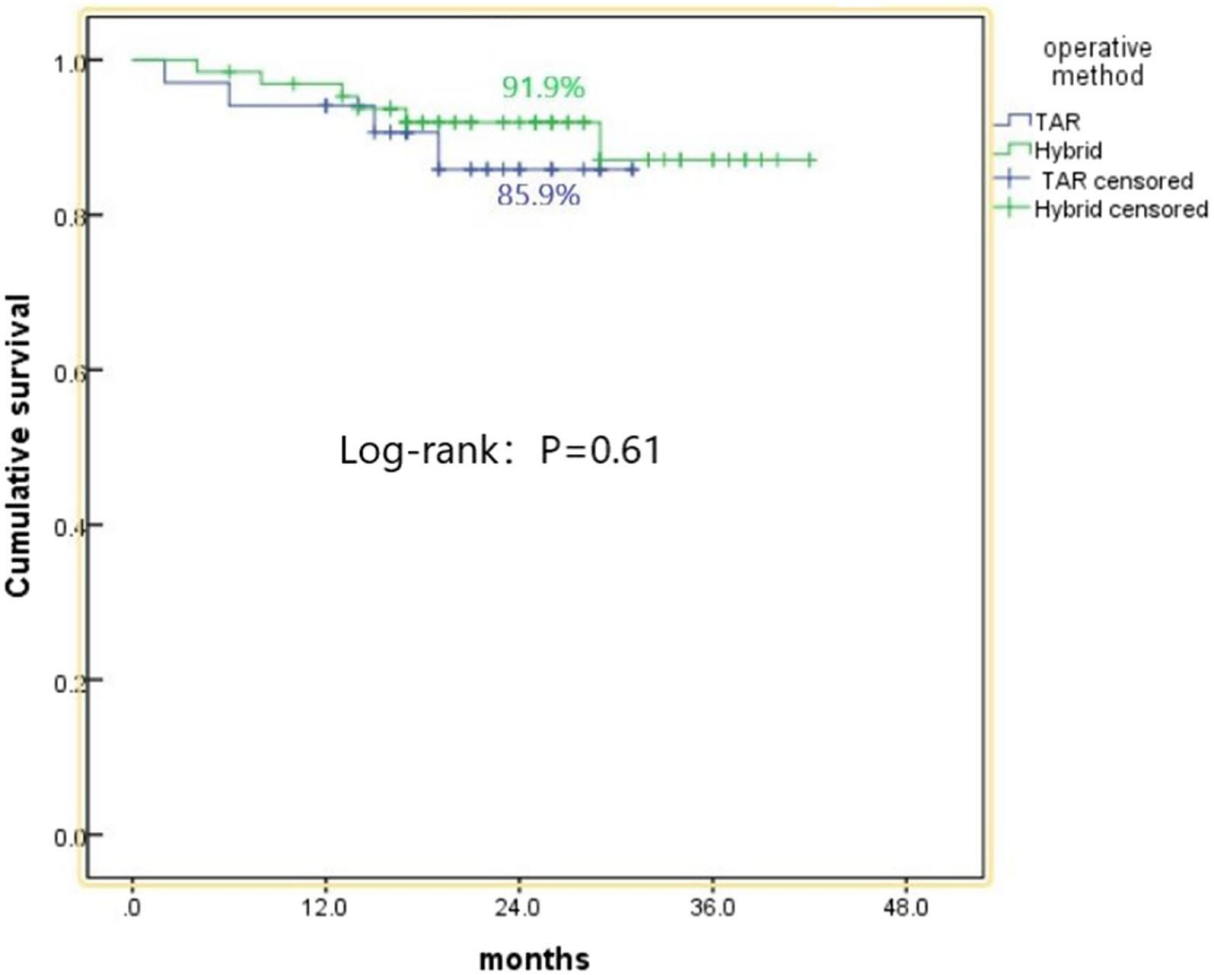
Survival function

The mean follow-up period was 26.1 months (24–42 months). Five patients lost to follow-up in the TAR group and one patient lost to follow-up in the hybridization group. The follow-up rates were 97.2% and 97.1%, respectively. During the follow-up period, there were twelve deaths in the TAR group. Four patients died of massive cerebral infarction, one died of gastrointestinal bleeding and two died in the reoperation. Five patients died suddenly from an unknown reason. In the hybridization group, there were five deaths, including one patient died of cerebral hemorrhage and four of an unknown reason. Kaplan Meier survival analysis showed that the survival rates at 2 years were 91.9% and 85.9%, in the TAR and hybrid groups, respectively (*P* = 0.61; Fig. 3). The 2 years follow-up data of the patients are shown in Table 4. Both before and after matching, there was no significant difference in the false lumen thrombosis state of the whole aorta,internal leakage and reoperation between the two groups.

**Table 4:** 2 years follow-up data

Variables	Total cohort (n = 147)			Propensity-matched cohort (n = 70)		
	TAR	Hybrid	P-value	TAR	Hybrid	P-value
	(n = 142)	(n = 35)		(n = 35)	(n = 35)	
Distal arch level^a^, n (%)	129 (90.8)	34 (97.1)	0.62	35 (100.0)	34 (97.1)	0.952
Distal edge of the ET prosthesis^a^, n (%)	87 (61.3)	16 (45.7)	0.138	18 (51.4)	16 (45.7)	0.931
Renal artery level^a^, n (%)	21 (14.8)	7 (20.0)	0.817	6 (17.1)	7 (20.0)	0.896
Terminal abdominal aorta level^a^, n (%)	13 (9.2)	1 (2.9)	0.205	0 (0)	1 (2.9)	0.321
Internal leakage, n (%)	13 (9.2)	3 (8.6)	0.914	1 (2.9)	3 (8.6)	0.310
Reoperation, n (%)	6 (4.2)	1 (2.9)	0.132	0 (0)	1 (2.9)	0.321

^a^False lumen thrombosis is defined as thrombosis of > 80% or complete obliteration of the false lumen

## Discussion

Acute aortic dissection is a clinical emergency with disastrous consequences, requiring urgent surgical treatment, because of the extremely high mortality rate associated with conservative management. There are three important branches of the aortic arch: the brachiocephalic trunk, the left common carotid artery, and the left subclavian artery. If the dissection involves them, it is difficult to treat clinically due to the particularity of its anatomical location. Therefore, the treatment of Acute type A aortic dissection has always been an important issue in aortic surgery research [6]. It generally has been acknowledged that the best treatment option for Acute type A aortic dissection is the total arch replacement combined with stented frozen elephant trunk implantation [7]. However, long time of cardiopulmonary bypass and hypothermic circulatory arrest are inevitable and regarded as the causes of complications and mortality [8]. With the aid of endovascular therapy concepts and devices, hybrid surgery had been applied for Stanford A aortic dissection (TAAD) repair in 2010 [9]. Hybrid surgery can combine vascular intervention with surgical treatment, reflect the advantages of interdisciplinary, provide a complete and systematic scientific therapy for patients. It is the latest trend in the development of treatment for acute aortic dissection [5]. Hybrid surgery avoids the deep hypothermic circulatory arrest and reduces the time of CPB. Compared with the traditional full-arch replacement surgery, the hybrid surgery has many advantages, such as short time, small trauma, and rapid postoperative recovery.

In a recent meta-analysis reported by Hsieh et al., compared with total arch replacement, hemiarch led to a significantly lower risk of in-hospital mortality [RR 0.77; 95% confidence interval (CI) 0.61–0.96] and a shorter CPB time (95% CI -56.68 to -49.50), circulatory arrest time (95% CI − 9.04 to 7.15) and antegrade cerebral perfusion time (95% CI -30.23 to -27.00), which is consistent with our results [10]. Several studies have shown that prolonged cardiopulmonary bypass is an independent risk factor for prolonged hospitalization in ICU and increased complications rate after cardiac surgery [11]. Deep hypothermic circulatory arrest in total arch replacement surgery is prone to coagulation dysfunction, which requires large amounts of blood transfusion. And blood transfusion also increases the risk of surgery [12]. The results of this study were consistent with those of previous studies. The blood consumption and cardiopulmonary bypass time of hybrid operation group were significantly less than those of total arch replacement group, which was beneficial to the recovery of patients and reduced complications related to cardiopulmonary bypass.

Deep hypothermic circulatory arrest increases the activation of inflammatory factors and aggravates ischemia–reperfusion injury, which is more damaging to the brain and other organs [13].The long-term deep hypothermic circulatory arrest will increase the incidence of neurological complications. Even short-term circulatory arrest also affects cognitive function. The longer the time of circulatory arrest, the higher the probability of brain complications and terminal organ damage. Previous studies regarding TAR procedure reported PND rates between 3 and 15% [14, 15]. In this study, the incidence of PND during hospitalization in TAR and hybrid groups was 5.6% and 0%, respectively (*P* = 0.152), which was similar to the findings of Smith et al. (3.7% vs 0). However, the incidence of TND in this study was 26.8% and 8.6%, respectively (*P* = 0.03). Therefore, the hybrid debranching could not reduce the incidence of cerebral hemorrhage, stroke and other severe neurological complications.

The perioperative mortality rates of the two groups were 8.5% and 2.9% (*P* = 0.268), and the 1-year survival rates were 96.6% and 94.1% (*P* = 0.61), respectively. There was no statistically significant difference between the two groups, which was similar to the results in the previous study [16, 17]. Therefore, the less invasive hybrid surgery makes the mortality rate of Acute type Aaortic dissection patients with high-risk indistinguishable from TAR. Consequently, we believe that debranching hybrid surgery is a safe and effective method for treating aortic dissection.

Although the advantages of hybrid surgery are apparent, it is currently mostly used in the elderly patients over 60 years old or in the patients who are not suitable for TAR surgery [9]. At present, the biggest problems of this procedure are the adhesiveness of the covered stent vessel at the aortic arch and the stent vessel service life. Covered stents have been used for more than 10 years in patients with type B dissection. However, the stent of B-type dissecting is mainly placed at the distal end of the arch and the descending aorta, while the A-type requires a stent to cover the entire aortic arch. Therefore, the pressure and shearing force endured by the stent in the two locations are not completely the same. As a new technique, the long-term effect of hybrid surgery cannot be clarified, which needs to be confirmed by a large number of clinical studies.

### Limitation

The main limitations of this study were a single-centre retrospective data and the short-term follow-up. In addition, the indications for the surgery methods were not uniform or standard.

## Conclusion

Hybrid debranching operation is a safe and effective method for Acute type Aaortic dissection. Compared with TAR surgery, hybrid debranching surgery has the characteristics of less trauma, rapid recovery and lower incidence of complication. Due to the lack of long-term follow-up data, the long-term efficacy of this technique needs to be further observed.

## Data Availability

Not applicable.

## Abbreviations

TAR: Total arch replacement
ICU: Intensive care unit
CTA: Computed tomography angiography
PT: Prothrombin time
INR: International standardized ratio
y: Years
mm: Millimeter
d: Day
h: Hour
min: Minute
u: Unit
ml: Milliliter
CBP: Cardiopulmonary bypass
ACC: Aortic cross-clamp
CA: Circulatory arrest
TND: Transient neurological dysfunction
PND: Permanent neurological dysfunction
TAAD: Stanford A aortic dissection
CI: Confidence interval
